# The Effects of Age, Cigarette Smoking, Sex, and Race on the Qualitative Characteristics of Lung Transcriptome

**DOI:** 10.1155/2020/6418460

**Published:** 2020-08-04

**Authors:** Qingzhou Guan, Juan Zhang, You Guo, Jie Xia, Jiahui Zhang, Jiajing Xie, Hao Cai, Haidan Yan, Xianlong Wang, Zheng Guo

**Affiliations:** ^1^Co-construction Collaborative Innovation Center for Chinese Medicine and Respiratory Diseases by Henan & Education Ministry of P.R. China, Academy of Chinese Medical Sciences, Henan University of Chinese Medicine, Zhengzhou 450046, China; ^2^Key Laboratory of Medical Bioinformatics, Key Laboratory of Ministry of Education for Gastrointestinal Cancer, School of Basic Medical Sciences, Fujian Medical University, Fuzhou 350001, China; ^3^The First Affiliated Hospital of Gannan Medical University, Ganzhou 341000, China

## Abstract

The within-sample relative expression orderings (REOs) of genes, which are stable qualitative transcriptional characteristics, can provide abundant information for a disease. Methods based on REO comparisons have been proposed for identifying differentially expressed genes (DEGs) at the individual level and for detecting disease-associated genes based on one-phenotype disease data by reusing data of normal samples from other sources. Here, we evaluated the effects of common potential confounding factors, including age, cigarette smoking, sex, and race, on the REOs of gene pairs within normal lung tissues transcriptome. Our results showed that age has little effect on REOs within lung tissues. We found that about 0.23% of the significantly stable REOs of gene pairs in nonsmokers' lung tissues are reversed in smokers' lung tissues, introduced by 344 DEGs between the two groups of samples (RankCompV2, FDR <0.05), which are enriched in metabolism of xenobiotics by cytochrome P450, glutathione metabolism, and other pathways (hypergeometric test, FDR <0.05). Comparison between the normal lung tissue samples of males and females revealed fewer reversal REOs introduced by 24 DEGs between the sex groups, among which 19 DEGs are located on sex chromosomes and 5 DEGs involving in spermatogenesis and regulation of oocyte are located on autosomes. Between the normal lung tissue samples of white and black people, we identified 22 DEGs (RankCompV2, FDR <0.05) which introduced a few reversal REOs between the two races. In summary, the REO-based study should take into account the confounding factors of cigarette smoking, sex, and race.

## 1. Background

Recently, we have revealed an important biological phenomenon that, despite high variations of gene expression levels among different individuals, the within-sample relative expression orderings (REOs) of genes are highly stable in a particular type of normal human tissue, which might be an intrinsic mechanism to keep genes functioning coordinately in the normal tissues. On the other hand, the stable REOs in the normal tissues are widely disturbed in diseased tissues [1, 2], providing abundant information for characterizing diseases [1, 3]. As the qualitative characteristics of transcriptomes, the within-sample relative expression orderings (REOs) of genes are highly robust against measurement variations and experimental batch effects [4–6]. Actually, taking these unique advantages of the REOs, some REO-based methods such as TSP [7], K-TSP [8] and others [9, 10] have been developed for discriminating cancer subtypes. Especially, many REO-based prognostic signatures have been proposed for specific medical issues for various cancers such as nonsmall cell lung cancer [3, 11], colorectal cancer [4, 12], and other cancers [13–15].

Based on the REOs analysis, we have proposed an algorithm named RankComp [1] to detect differentially expressed genes (DEGs) for an individual disease sample compared with its previous normal state through analyzing which genes' up- or downregulation may lead to the reversal REOs in the disease sample, taking the stable REOs predetermined in a large collection of the normal tissue samples as the normal background [1, 16]. The individual-level analysis of DEGs allows us to identify subtype-specific genes, which can provide us novel perspectives for understanding the mechanisms of carcinogenesis [16]. In contrast, for a DEG detected at the population-level, we cannot know whether it is differentially expressed in a particular cancer sample because of the heterogeneity of cancer. The REOs analysis method could also be applied to the identification of disease-associated genes or pathways based on one-phenotype disease data when the normal tissues are unavailable or insufficient for some vital organs such as the brain and heart [1, 17–19]. In this situation, it is of great value to reuse the normal control data accumulated in other studies. And we have proposed a REO-based algorithm, named DRFunc [19], to identify disease-associated pathways based on one-phenotype data through comparing the stable REO in the one-phenotype disease samples with the normal stable REOs background predetermined in previously accumulated normal samples from other studies. Based on the REOs analysis, we have also proposed a method named “RankCompV2” for identifying DEGs at the population-level through comparing the stable REOs of two phenotypes [20].

The above mentioned differential expression analysis methods based on REO comparisons are all dependent on the normal stable REOs background predetermined from previously accumulated normal samples. However, some confounding factors such as age, cigarette smoking, sex, and race may affect the gene expression levels in normal samples. Studies have shown that sex-biased gene expression is widespread across genomes on both sex chromosomes and autosomes [21, 22]. Several studies have also reported that cigarette smoking [23] and race [24] could alert the gene expression levels, and the gene expression levels change with age in many organ tissues, including lung tissues [25]. However, whether those confounding factors could affect the REO of gene pairs is still unknown.

Thus, in this paper, using the normal lung tissue samples from three different laboratories, we evaluated the effects of four confounding factors, including age, cigarette smoking, sex, and race, on the REOs within normal lung tissues.

## 2. Methods

### 2.1. Data and Preprocessing

The gene expression profiles analyzed in this study are described in Table 1. All the datasets were measured by the Affymetrix GPL570 platform, and the processed data were directly downloaded from the Gene Expression Omnibus database. For the downloaded data, each probe ID was mapped to Entrez gene ID with the corresponding platform file. If a probe was mapped to multiple or zero genes, the data were discarded. If multiple probes were mapped to the same gene, the expression value of the gene was defined as the arithmetic mean of the values of these probes. Notably, nonsmokers included in this study denote individuals without the history of cigarette smoking.

### 2.2. Evaluation of Confounding Factors on REO of Gene Pairs

Within a sample, the REO of two genes, A and B, is denoted as A > B (or B < A) if the expression level of gene A is higher (or lower) than that of gene B. For each of the three binary confounding factors, cigarette smoking, sex, and race, we first divided the samples into two groups and then identified the gene pairs with significantly stable REOs in each of the group. The significance of a gene pair with stable REO in a group of samples was determined by the binomial test as follows:

(1) $$\begin{array}{c} p=1-\overset{k-1}{\underset{i=0}{\sum }}\left(\begin{array}{c} n\\ i \end{array}\right)p_{0}^{i}{\left(1-p_{0}\right)}^{n-i} \end{array}$$

where the REO pattern (A > B or A < B) is consistent among *k* samples out of *n* samples in total and *p*_0_ (*p*_0_ = 0.5) is the probability of observing one of two possible REO outcomes in a sample by chance. The *P*-values were then adjusted using the Benjamini and Hochberg method [26].

A gene pair with stable REOs in both groups of samples but the REO directions are opposite is called a reversal gene pair. Otherwise, if the REO directions are consistent in both groups, it is called a concordant gene pair. If the two lists of stable gene pairs identified above have *m* common pairs, among which *k* have opposite REO directions, the reversal ratio is calculated as *k*/*m*.

Between the two groups of samples classified by a binary confounding factor, the distribution of other confounding factors between the two groups was tested by the Fisher exact test to ensure that there is no significant difference for the other confounding factors. For the age factor, the samples were divided into two groups based on the REO pattern of each gene pair, and then, we test whether there is a significant difference in age between the two groups of samples based on the Mann–Whitney *U*-test. The REO of the gene pair is significantly correlated with age if the age is significantly different between the two groups.

### 2.3. Identification of Differentially Expressed Genes

Focusing on the stable gene pairs commonly identified from two groups of samples, we identified the concordant and the reversal REOs between the two groups for a specific factor. RankCompV2 [20] was applied to detect differentially expressed genes (DEGs) between the two groups of samples. The details of the RankCompV2 algorithm has been described in ref. [20]. Briefly, Fisher's exact test was applied to identify whether a gene may disrupt the gene correlation structure in one group compared to the other group based on the concordant and the reversal REOs between the two groups. For a particular gene, to minimize the potential effect of other genes' expression changes on the Fisher's exact test, an iterative filter process [27] was conducted.

### 2.4. Pathway Enrichment Analysis

Data of 238 pathways covering 6638 unique genes were extracted from the Kyoto Encyclopedia of Genes and Genomes (KEGG) on May 3, 2017. The hypergeometric distribution model was used to determine the significance of biological pathways enriched with up- and downregulated DEGs, respectively.

## 3. Results

### 3.1. The Influence of Age on REOs within Normal Lung Tissues

From three datasets (GSE31210, GSE19804, and GSE20257, as shown in Table 1), we selected 65 samples of nonsmoking Asian females with age ranging from 37 to 80 years old for the analysis. Based on the REO pattern of each gene pair, the samples were divided into two groups, and then, the Mann–Whitney *U*-test was used to test whether there is significant difference in age between the two groups of samples. We could not find any gene pair whose REO was significantly correlated with age with FDR <0.05 or even with FDR <0.2 (Methods). Similarly, using 34 samples for Caucasian males with age ranging from 27 to 80 years old, collected from the dataset GSE4115, no significant gene pair was found with either FDR <0.05 or FDR <0.2.

The above results indicated that the influence of age on REO of gene pair could be negligible. Accordingly, the age factor was not taken into account in the subsequent analyses.

### 3.2. The Influence of Cigarette Smoking on REOs within Normal Lung Tissues

We compared the gene expression profiles of normal lung tissue samples for 49 smokers and 44 nonsmokers from the GSE20257 dataset. The detailed information on the sample composition was shown in Table 2. There is no significant difference in sex or race distribution between the smoker group and the non-smoker group (Fisher's exact test, *P* > 0.1).

**Figure 1:**
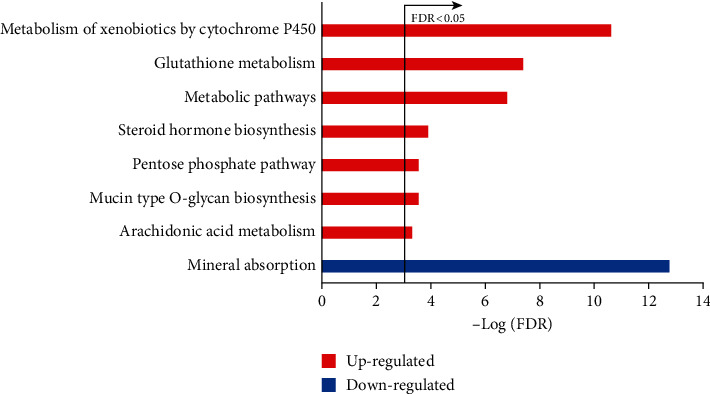
The KEGG pathways separately enriched with up- and downregulated genes in the smoker group compared with the nonsmoker group.

With FDR <0.05, we identified the gene pairs with significantly stable REOs in the smoker group and nonsmoker group, respectively. We found 187,875,560 gene pairs that have significantly stable REOs (binomial test, FDR <0.05) in both groups, among which 0.227% showed reversal REO patterns. With RankCompV2, we identified 344 DEGs, including 210 up- and 134 downregulated genes in the smoker group compared with the non-smoker group (FDR <0.05). The 210 upregulated genes and 134 downregulated genes were enriched, respectively, in 7 pathways and 1 pathway (hypergeometric test, FDR <0.05), as shown in Figure 1. For the pathway “metabolism of xenobiotics by cytochrome P450”, cytochromes P450 are known to be responsible for the metabolism of compounds present in cigarette smoke, including nicotine, benzene, polycyclic aromatic hydrocarbons (PAHs), and tobacco-specific nitrosamines (TSNAs) [28]. As for the “glutathione metabolism” pathway, it has been found that cigarette smoking could induce the deregulation of glutathione metabolism in bronchial epithelial cells [29]. It has also been reported that “metabolic pathways,” [30] “steroid hormone biosynthesis,” [31] “pentose phosphate pathway,” [32] “arachidonic acid metabolism,” [33] and “mineral absorption” [34] are affected by cigarette smoking.

The above results indicated that cigarette smoking can alter the REOs in normal lung tissues and disturb some important biological pathways.

### 3.3. The Influence of Sex on REOs within Normal Lung Tissues

We compared the gene expression profiles of normal lung tissue samples for 64 males and 29 females from the dataset GSE20257. The detailed information of the sample composition was shown in Table 3. There is no significant difference in smoking rate or race distribution between the male group and the female group (Fisher's exact test, *P* > 0.2).

We identified the gene pairs with significantly stable REOs in the male and female groups, respectively, and found 187,481,246 gene pairs with significantly stable REOs (binomial test, FDR <0.05) in both groups, among which 0.074% showed the reversal REO patterns. With RankCompV2, we identified 35 DEGs in the male group compared with the female group (FDR <0.05). In another dataset GSE71181, including 201 male samples and 80 female samples which are all from smokers, 25 of the above 35 DEGs were also found (*T*-test, FDR <0.05) and 96% (24 genes) have the same dysregulation directions in the male group compared with the female group. Among the 24 DEGs, 6 out of the 10 upregulated genes in the male group are located on Y chromosome, 12 out of the 14 upregulated genes in the female group are located on X chromosome, and the cytoband of these genes is shown in Table 4. In particular, DDX43, CRISP2, and PRDM7, which are upregulated in the male group, are located on autosome and involved in spermatogenesis and male fertility [35, 36]. For the other two genes, NLRP2 and C3orf79, located on autosome but upregulated in the females, it is known that NLRP2 is a critical regulator of oocyte [37].

### 3.4. The Influence of the Race Factor on REOs within Normal Lung Tissues

Due to the limitation of the sample sizes for other races, we only compared the gene expression profiles of normal lung tissues for the white and black races. From the GSE20257 dataset, we obtained 34 samples for white people and 59 samples for black people. The detailed information of the sample composition was shown in Table 5. There is no significant difference in cigarette smoking rate or sex distribution between the two groups (Fisher's exact test, *P* > 0.1).

**Figure 2:**
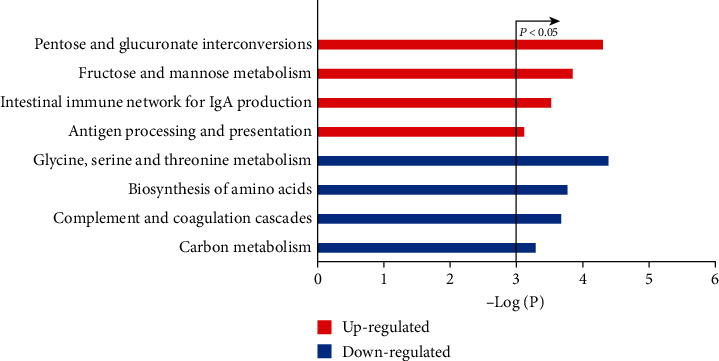
The KEGG pathways separately enriched with up- and downregulated genes in the white people compared with the black people.

With FDR <0.05, we found 187,973,147 gene pairs with significantly stable REOs in both groups, among which 0.0272% showed reversal REO patterns. With RankCompV2, we identified 22 DEGs, including 10 up- and 12 downregulated genes in the white group compared with the black group (FDR <0.05). Due to the small number of DEGs, we found no pathway significantly enriched with the up- or downregulated DEGs with FDR <0.05. With *P* < 0.05, the 10 upregulated and 12 downregulated genes were enriched in, respectively, 4 and 4 pathways, as shown in Figure 2. The result indicates that there are some differences in metabolism and immunity of the normal lung tissues between the white and black races [38, 39].

## 4. Discussion

Among the four confounding factors investigated in this paper, cigarette smoking alters the REOs within lung tissues most widely, and sex and race can also alter the REOs but only slightly, whereas there is no evidence that age could affect the REO of gene pairs. Therefore, the REO-based study should take into account the confounding factors of cigarette smoking, sex, and race. When building the normal stable REOs background based on previously accumulated normal samples from other studies, the normal samples should include sufficient samples with the same factors presenting in the one-phenotype disease samples analyzed in a study.

Our results showed that cigarette smoking disrupts “metabolism of xenobiotics by cytochrome P450,” “glutathione metabolism,” and other pathways [28, 29], and there are some differences in metabolism and immunity between different races. The sex factor affects some genes located on the sex chromosome and some genes located on the autosomes which are involved in spermatogenesis, male fertility [35] and are critical regulator of oocyte [37]. Because cigarette smoking, sex, and race could affect the REO of gene pairs, the influence of these factors should be taken into account in the REO-based analysis for lung tissue.

This study exists some limitations. Due to the limitation of normal tissue samples and clinic information for many other organs, we only systematically analyze the influence of the four common confounding factors (age, cigarette smoking, sex, and race) on REOs in the normal lung tissues. The effects of the confounding factors on the REOs might be tissue specific. We have primarily analyzed the influence of sex on REOs of gene pairs in normal stomach tissues and esophagus tissues, respectively, and found that all the DEGs are located on sex chromosome, as described in Supplementary files 1. Future studies on the effect of confounding factor on the REOs of gene pairs in tissues of other organs need to be further studied.

## 5. Conclusions

Our results show that the confounding factors, including cigarette smoking, sex, and race could alter the REOs within lung tissues. Thus, the REO-based study should consider these confounding factors. Moreover, the effect of age on REO of gene pair could be negligible.

## Figures and Tables

**Table 1:** Data used in this study.

Characteristics	GEO Acc	GSE31210	GSE19804	GSE20257
	Sample size	20	60	93
Age	Median	59 (30–89)	61 (37–80)	45 (21–73)
Smoking history	Smoker	12	/	49
	Nonsmoker	8	60	44
Sex	Male	11	/	64
	Female	9	60	29
Race	Asian	20	60	/
	White	/	/	34
	Black	/	/	59

Notes: “/” cells indicate that there is no sample in the corresponding category.

**Table 2:** The data extracted from GSE20257 for the analysis of the cigarette smoking factor.

Characteristics	Smoker (*n* = 49)	Nonsmoker (*n* = 44)	Fisher's exact test
Sex			*P* = 0.266
Male	31	33	
Female	18	11	
Race			*P* = 0.131
White	14	20	
Black	35	24	

**Table 3:** The data extracted from GSE20257 for the analysis of the sex factor.

Characteristics	Male (*n* = 64)	Female (*n* = 29)	Fisher's exact test
Race			*P* = 0.495
White	25	9	
Black	39	20	
Smoking history			*P* = 0.266
Smoker	31	18	
Nonsmoker	33	11	

**Table 4:** The cytoband of the 24 sex-biased genes.

Upregulated genes in males		Upregulated genes in females	
Symbol	Cytoband	Symbol	Cytoband
TTTY10	Yq11.221	SMC1A	Xp11.22-p11.21
PRKY	Yp11.2	DDX3X	Xp11.3-p11.23
TBL1Y	Yp11.2	STS	Xp22.32
KDM5D	Yq11	RIBC1	Xp11.22
DDX3Y	Yq11	ZFX	Xp21.3
UTY	Yq11	EFHC2	Xp11.3
ARSE	Xp22.3	KDM6A	Xp11.2
PRDM7	16q24.3	JPX	Xq13.2
DDX43	6q13	ZRSR2	Xp22.1
CRISP2	6p12.3	PNPLA4	Xp22.3
		ARSD	Xp22.3
		GEMIN8	Xp22.2
		C3orf79	3q25.2
		NLRP2	19q13.42

**Table 5:** The data extracted from GSE20257 for the analysis of the race factor.

Characteristics	White (*n* = 34)	Black (*n* = 59)	Fisher's exact test
Sex			*P* = 0.495
Male	25	39	
Female	9	20	
Smoking history			*P* = 0.131
Smokers	14	35	
Nonsmokers	20	24	

## Data Availability

The data used to support the findings of this study are available from the corresponding author upon request.

## Abbreviations

REO: Relative expression orderings
DEGs: Differentially expressed genes.

## Supplementary Materials

Supplementary Materials Table S1: the DEGs list between male and female samples for normal stomach tissues. Table S2: the DEGs list between male and female samples for normal esophagus tissues.
