# Supplementary material for: The Effects of Age, Cigarette Smoking, Sex, and Race on the Qualitative Characteristics of Lung Transcriptome
Source: Biomed Res Int. 2020 Aug 4;2020:6418460. doi: 10.1155/2020/6418460 (PMC7424369; doi:10.1155/2020/6418460)
Supplement: Supplementary Materials — Table S1: the DEGs list between male and female samples for normal stomach tissues. Table S2: the DEGs list between male and female samples for normal esophagus tissues. [file 6418460.f1.docx]

**Supplementary Results**

**The influence of sex on REOs within normal stomach and esophagus tissues**

In addition, using 23 male and 12 female normal stomach tissue samples from TCGA, we also evaluated the influence of sex on REOs of gene pairs. With FDR<0.05, the significantly stable gene pairs were identified in male and female groups respectively. 168, 213, 439 gene pairs were commonly identified in the two groups, among which 0.071% showed the reversal REO patterns. Then, using the RankCompV2, 16 DEGs were identified between the male and the female groups (FDR<0.05), the DEGs list was shown in Table S1. And all the DEGs were located on sex chromosome, 15 up-regulated genes in the male group compared with the female group were located on Y chromosome, whereas 1 up-regulated gene in the female group compared with the male group was located on X chromosome, which indicated that sex could also slightly affect the REO of gene pairs in the normal stomach tissue samples.

**Table S1. The DEGs list between male and female samples for normal stomach tissues**

| **Up-regulated in male** | | **Up-regulated in female** | |
| --- | --- | --- | --- |
| **Symbol** | **Cytoband** | **Symbol** | **Cytoband** |
| TXLNGY | Yq11.222 | TSIX | Xq13.2 |
| CYorf15B | Yq11.222 |  |  |
| DAZ1 | Yq11.223 |  |  |
| DDX3Y | Yq11 |  |  |
| EIF1AY | Yq11.223 |  |  |
| KDM5D | Yq11 |  |  |
| NCRNA00185 | Yq11.222 |  |  |
| NLGN4Y | Yq11.221 |  |  |
| PRKY | Yp11.2 |  |  |
| TMSB4Y | Yq11.221 |  |  |
| TTTY14 | Yq11.222 |  |  |
| TTTY15 | Yq11.1 |  |  |
| USP9Y | Yq11.2 |  |  |
| UTY | Yq11 |  |  |
| ZFY | Yp11.3 |  |  |

Similarly, for normal esophagus tissue, 69 male samples and 17 female samples were collected from 6 datasets (GSE3526, GSE7307, GSE45670, GSE17351, GSE36223 and gse39491). With FDR<0.05, the significantly stable gene pairs were identified in male and female groups respectively. 65, 690, 678 gene pairs were commonly identified in the two groups, among which 0.081% showed the reversal REO patterns. With RankCompV2, we identified 12 DEG between the male group and the female group (FDR<0.05), the DEGs list was shown in Table S2. And all the DEGs were located on sex chromosome, 10 up-regulated genes in the male group compared with the female group were located on Y chromosome, whereas 2 up-regulated genes in the female group compared with the male group were located on X chromosome, which indicated that sex could also slightly affect the REO of gene pairs in the normal esophagus tissue samples.

**Table S2. The DEGs list between male and female samples for normal esophagus tissues**

| **Up-regulated in male** | | **Up-regulated in female** | |
| --- | --- | --- | --- |
| **Symbol** | **Cytoband** | **Symbol** | **Cytoband** |
| PRKY | Yp11.2 | PRKX | Xp22.3 |
| RPS4Y1 | Yp11.3 | XIST | Xq13.2 |
| UTY | Yq11 |  |  |
| ZFY | Yp11.3 |  |  |
| KDM5D | Yq11 |  |  |
| USP9Y | Yq11.2 |  |  |
| DDX3Y | Yq11 |  |  |
| EIF1AY | Yq11.223 |  |  |
| TTTY15 | Yq11.1 |  |  |
| TXLNGY | Yq11.222 |  |  |
